# Regulation of VDAC trafficking modulates cell death

**DOI:** 10.1038/cddiscovery.2016.85

**Published:** 2016-12-12

**Authors:** Ashvini K Dubey, Ashwini Godbole, M K Mathew

**Affiliations:** 1 Laboratory of Membrane Biophysics, National Centre for Biological Sciences, TIFR, Bangalore 560065, India; 2 Department of Crop Physiology, GKVK, University of Agricultural Sciences, Bangalore 560065, India

## Abstract

The voltage-dependent anion channel (VDAC) and mitochondria-associated hexokinase (HxK) have crucial roles in both cell survival and death. Both the individual abundances and their ratio seem to influence the balance of survival and death and are thus critical in scenarios, such as neurodegeneration and cancer. Elevated levels of both VDAC and HxK have been reported in cancerous cells. Physical interaction is surmised and specific residues or regions involved have been identified, but details of the interaction and the mechanism by which it modulates survival are yet to be elucidated. We and others have shown that heterologous expression of VDAC can induce cell death, which can be mitigated by concomitant overexpression of HxK. We have also observed that upon overexpression, fluorescently tagged VDAC is distributed between the cytosol and mitochondria. In this study, we show that cell death ensues only when the protein, which is synthesized on cytoplasmic ribosomes, migrates to the mitochondrion. Further, coexpression of rat HxK II (rHxKII) can delay the translocation of human VDAC1 (hVDAC1) protein to mitochondria and thereby inhibit VDAC-induced cell death. Variation in the level of HxK protein as seen endogenously in different cell lines, or as experimentally manipulated by silencing and overexpression, can lead to differential VDAC translocation kinetics and related cell death. The N-terminal region of HxK and the Glu73 residue of hVDAC1, which have previously been implicated in a physical interaction, are required for cytosolic retention of VDAC. Finally, we show that, in otherwise unperturbed cells in culture, there is a small but significant amount of soluble VDAC in the cytosol present in a complex with HxK. This complex could well determine how a cell is poised with respect to incoming thanatopic signals, thereby tilting the survival/death balance in pharmacologically interesting situations, such as neurodegeneration and cancer.

## Introduction

The voltage-dependent anion channel (VDAC) is the most abundant protein in the mitochondrial outer membrane, with critical roles in several cellular processes, such as mitochondrial bioenergetics, calcium signaling and cell death.^1–5^ Regulation of VDAC function in these diverse processes is critical and has become a subject of intense investigation. The channel consists of a *β* barrel with a single nanometer-sized pore that serves as the principal conduit between cytosol and mitochondrion.^6^ Mammalian mitochondria have three VDACs of which VDAC1 is by far the most abundant and well studied. VDAC2 and VDAC3 have distinct functions, including an antiapoptotic role for VDAC2.^7^ We have focused on VDAC1, which has been clearly implicated in cell death processes in a proapoptotic role.

Although the protein is named for the voltage dependence of its conductance when reconstituted into planar bilayers, all documented modulation of its function under physiological conditions appears to be ascribable to its interaction with other proteins. A variety of molecules, including cytoskeletal proteins, IP3R, Bcl2 family proteins, mitochondrial membrane proteins (for example ANT) and mitochondrial localized kinases (hexokinase (HxK) and creatine kinase), have been shown to interact with VDAC and control its function.^8–11^

The interplay of HxKII with VDAC1 in cellular signaling in cancer and neurodegeneration has attracted considerable attention over the past decade.^2,3,12^ In particular, a role for the two proteins has been suggested in the much discussed Warburg Effect,^9^ making these proteins and their interactions targets for novel drug development.^13^ There are three isoforms of HxK in mammalian cells, all of which add a phosphate group to glucose at the sixth position. This serves, on the one hand, to retain freshly imported glucose as a charged moiety within the cell. On the other hand, it is also the entry point into glycolysis and the pentose phosphate pathway. Cancerous cells have highly elevated levels of glycolysis compared with surrounding healthy tissue, even when oxygen is not limiting (the Warburg Effect^9,14^) and HxKII has been shown to have a major role in this switch. Apart from these roles in metabolism, mitochondrial isoforms of HxK, HxKI and HxKII, have been implicated in the regulation of cellular death processes.^15^

Survival *versus* death outcomes in plant as well as animal cells, including neurons and cancer cells, have been shown to be modulated by VDAC–HxK interactions.^16–18^ Previous studies from our^18^ as well as other^2,10^ laboratories have shown that elevation in the expression of both VDAC and HxK is associated with increased growth rate as seen in cancerous cells exhibiting the Warburg Effect,^9^ whereas increased expression of any one of the proteins tilts the balance of cell survival and death.^10,18^ Increased expression of HxK alone makes cells resistant to cell death.^19,20^ This protective effect of overexpression of HxK is limited in the absence of VDAC as shown in VDAC-silenced human dopaminergic SH-SY5Y neuroblastoma cells.^17^ On the other hand, overexpression of VDAC alone leads to cell death,^21,22^ which can be prevented by coexpression of proteins, such as HxK and Bcl-2.^1,9,21,22^

Cell death resulting from overexpression of VDAC could be ameliorated by VDAC inhibitors, such as 4,4′-diisothiocyano-2,2′-disulfonic acid stilbene (DIDS) and Ruthenium Red (RuR).^18,21,22^ The mechanism of amelioration remains to be elucidated. Zaid *et al.* have shown that, in planar bilayer membranes, HxK and RuR interact with VDAC directly, leading to decreases in VDAC conductance.^22^ These studies identified residues in VDAC (E72, D77 and E202), mutations in which abrogate the ability of HxK and RuR to modulate both VDAC conductance in artificial membranes and VDAC-mediated death cascades in cells.^22,23^ The studies imply that interaction with the modulators stabilizes the ‘Closed’ state of VDAC and thereby inhibit cell death. Although the ‘Open’ conformation of VDAC, populated when the electric field across the membrane is close to zero, allows passage of ATP and ADP, its ‘Closed’ conformation, favoured by high electric fields, allows permeation of cations, such as Ca^++^ but not ATP and ADP.^24,25^ The simple-minded assumption would be that the ‘Open’ conformation is essential for survival of cells, whereas conditions favouring the ‘Closed’ state would promote cell death. Data from several groups suggest that antiapoptotic proteins Bcl2 and BclXL maintain the Open conformation of VDAC and thereby promote survival.^26,27^ If both sets of modulators function by biasing the ‘Open *versus* Closed’ distribution of VDAC, then these two sets of studies would appear to be contradictory. Further, the experimental demonstration that mutation of the VDAC residues E72, D77 and E202 eliminates the protective effect of HxK appears to be in conflict with the reported 3D structures of VDAC.^28–30^ These structures place some of the residues (for example, E72) shown to be critical for interaction with HxK and RuR within the transmembrane portion of the *β* barrel and thus inaccessible to proteins as large as HxK. HxKII is present both in the cytosol and mitochondria and is presumed to interact with VDAC1 on the mitochondrion for its antiapoptotic effect. Both glucose phosphorylation activity as well as mitochondrial binding of HxK have been associated with its antiapoptotic function.^31^ The N-terminal truncated form of HxK, which remains exclusively in the cytosol, has also been reported to inhibit apoptosis albeit to a reduced extent.^31^ It is unclear how inhibition of cell death is brought about in this case.

It is clear that several gaps exist in the understanding of VDAC–HxK interactions – both in the normal physiology of the cell and in pathological conditions. Given the central role of this interaction in critical processes such as cell death and the Warburg Effect, we have taken a closer look at the antiapoptotic effects of HxK on VDAC-mediated cell death. Examining the consequences of natural and experimentally manipulated variation in the levels of expression of HxK and VDAC suggests that the distribution of VDAC between the cytosol and mitochondrion is subject to regulation by HxK. Mutating sites of interaction between the two proteins establishes this link and suggests that regulated retention of VDAC in the cytosol may have a role in the modulation of mitochondrially mediated cell death.

## Results

### Correlation of VDAC localization and cell death

**Figure 1:**
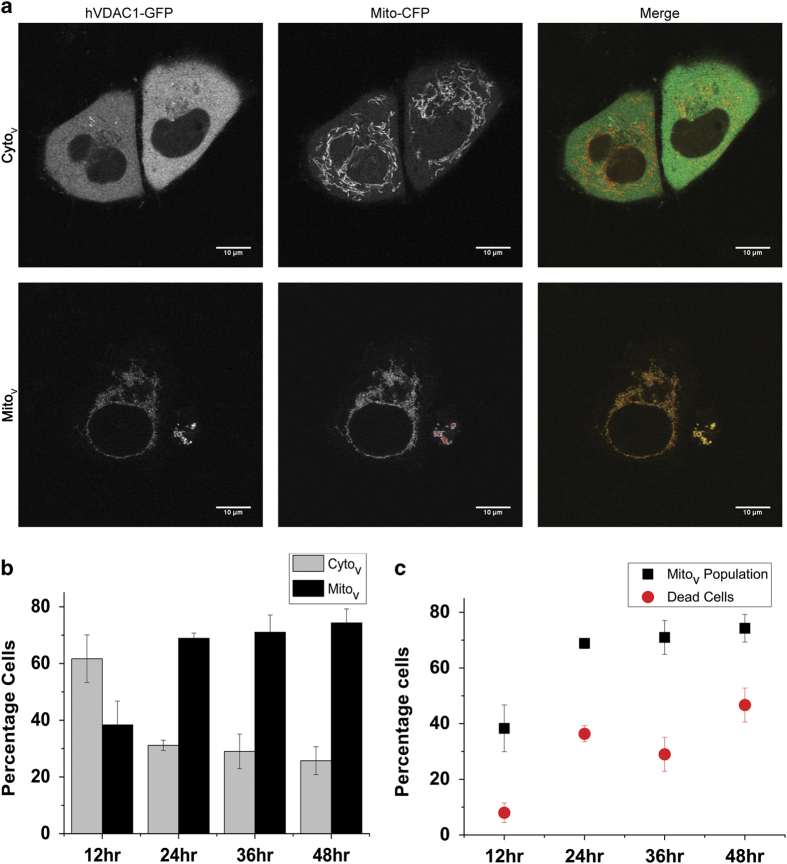
Cell death induced by hVDAC1-GFP and its distribution. (**a**) Representative images of HeLa cells with hVDAC1-GFP distributed through the cytosol (Cyto_v_, upper row) or localized to mitochondria (Mito_v_, lower row). Mitochondria were marked with CFP (mito-CFP). (**b**) Intracellular distribution of hVDAC1-GFP at different time points after transfection. (**c**) Relationship between mitochondrial localization of hVDAC1-GFP and the percentage of cell death at different posttransfection time points.

In this study, we expressed GFP-tagged human VDAC1 (hVDAC1-GFP) in HeLa cells and monitored both VDAC localization and cell death as a function of time. As seen earlier in Jurkat cells, VDAC in HeLa cells was also found to be either diffusely distributed through the cytosol or present in mitochondria (Figure 1a), with very few instances where it was present in both the compartments. The population of HeLa cells exhibiting mitochondrially localized VDAC protein (Mito_v_) increased from 37% at 12 h after transfection to >70% at 48 h (Figure 1b). Cell death, as monitored by nuclear fragmentation, also increased from <10% at 12 h to 40% at 24 h (Figure 1c). At both time points, death was restricted to cells where the protein was localized to mitochondria. We used mitochondria-targeted CFP (mito-CFP) to monitor mitochondrial morphology and found that the organelles were tubular and well spread in cells where VDAC was cytoplasmic (Cyto_v_) but were clumped and restricted to the perinuclear space in Mito_v_ cells (Figure 1a).

### Antiapoptotic proteins control VDAC localization

**Figure 2:**
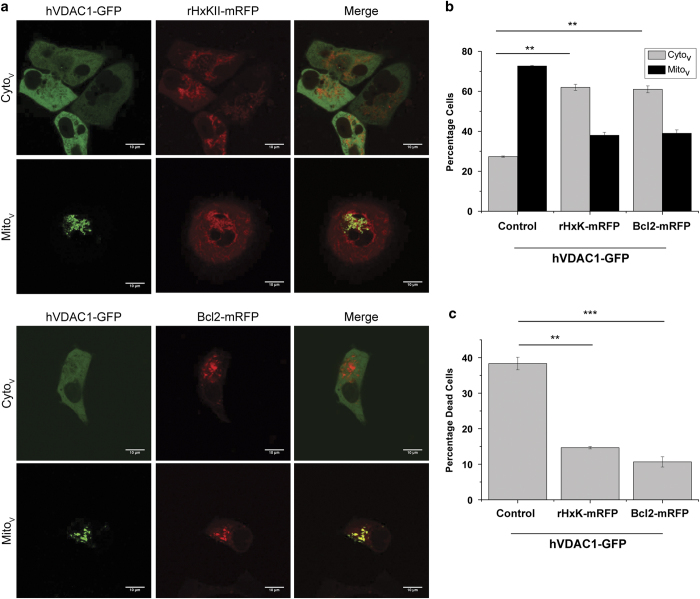
Regulation of VDAC localization by antiapoptotic proteins, HxK and Bcl2. (**a**) Representative images of cells of hVDAC1-GFP located in the cytosol or in mitochondria in cells coexpressing rHxKII-mRFP (upper panel) or Bcl2-RFP (lower panel). (**b**) Percentage of cells in the two populations shown in panel **a**. (**c**) Quantification of cell death in cells expressing hVDAC1-GFP either alone or together with rHxKII-mRFP or Bcl2-RFP. Data in panels **b** and **c** represent mean and S.E. of four experiments, with around 100 cells scored in each experiment. ***P=0.001, **P<0.005 based on Student’s unpaired *t*-test.

HxKII and Bcl-2 have been shown to attenuate the extent of cell death induced by heterologous VDAC expression.^10^ A dramatic shift in the distribution of hVDAC1-GFP was seen on coexpression with RFP-tagged HxKII or Bcl-2. hVDAC1-GFP was cytoplasmically localized in over twice as many cells coexpressing rat HxKII (rHxkII–mRFP) (62%) or Bcl2-mRFP (61%) as compared with cells expressing VDAC alone (27%) after 24 h of transfection (Figures 2a and b). Coexpression of HxKII or Bcl2 reduced cell death to 14% and 10%, respectively; whereas it was 38% in cells expressing only hVDAC1 (Figure 2c).

### Effect of HxKII expression level on VDAC localization and corresponding cell death

The obverse of the overexpression studies presented above is to reduce levels of endogenous HxK, which we have carried out using RNA interference. In addition, we have examined the behavior of heterologously expressed VDAC in cell lines that vary in their endogenous expression of HxK.

#### HxKII silencing

We used RNAi constructs (shHxKII) specific to HxKII in order to reduce the level of HxKII expression in HeLa cells without directly affecting the expression levels of other isoforms. As monitored by western blotting, the levels of HxKII protein reduced significantly within 12 h of transfection with shHxKII (Supplementary Figures S1A and B). Vector-borne GFP was used as a marker for the cells that are transfected with the HxKII RNAi construct. We used mCherry-tagged hVDAC1 (hVDAC1-mCherry) to monitor VDAC localization in these experiments with shHxKII.

**Figure 3:**
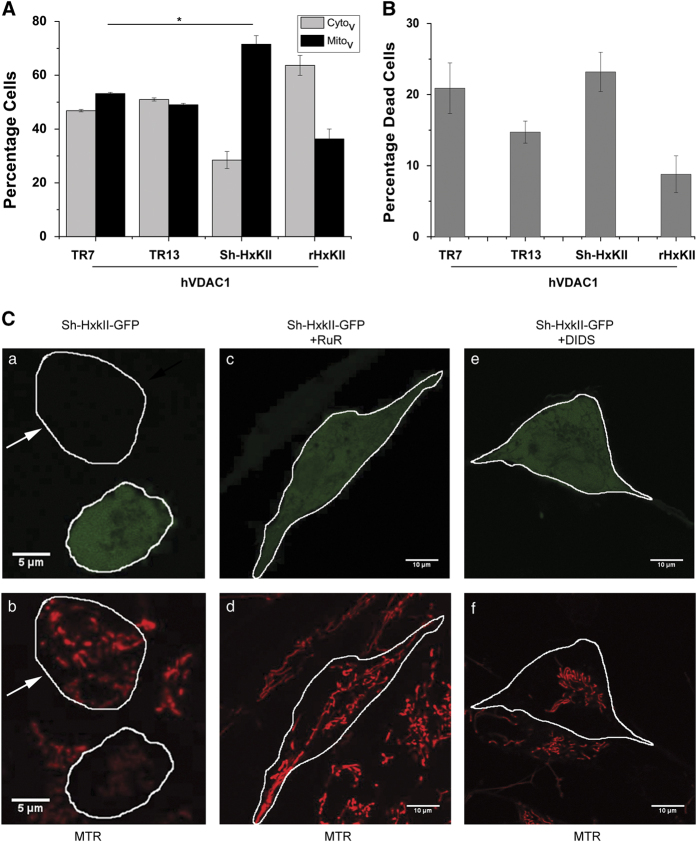
Effect of HxKII silencing on hVDAC1-mCherry localization, cell death and mitochondrial membrane potential. (**A**) Percentage of HeLa cells displaying cytosolic or mitochondrial localization of hVDAC1-mCherry 20 h after transfection with different RNAi constructs. Cells were cotransfected with hVDAC1-mCherry and either control plasmid (TR7) or scrambled plasmid (TR13) or silencing RNAi construct (shHxKII). (**B**) Percentage cell death in the groups shown in panel a. Data represent mean and S.E. of four independent experiments, with ≥100 cells were scored in each experiment. *P<0.01 based on Student’s unpaired *t*-test. (**C**) Representative images showing that HxKII silencing induced loss of mitochondrial membrane potential (a, b) and protection by VDAC inhibitors, (c, d) RuR and (e and f) DIDS. The cells expressing GFP indicating transfection with the silencing construct are marked by white solid outlines (a, c and e). Mitochondrial membrane potential was monitored by staining with MTR dye (b, d and f). Cells transfected with HxK silencing construct show reduced mitochondrial membrane potential (marked with white line in panels a and b), while an untransfected cell in the same field shows unpurturbed mitochondrial potential as indicated by bright MTR staining (cell marked with white arrow in panels a and b).

Cells expressing hVDAC1 are equipoised at 20 h after transfection, with both Cyto_v_ and Mito_v_ around 50%. Almost half the Cyto_v_ fraction shifts to the Mito_v_ fraction in cells treated with shHxKII (Figure 3A and Supplementary Figure S1B). At this time point, HxKII-silenced cells overexpressing hVDAC1-mCherry showed a marginal increase in the percentage of dead cells as compared with cells transfected with RNAi vector control and hVDAC1-mCherry (Figure 3B). In similar conditions overexpression of HxKII can reduce both the percentage of Mito_v_ cells (Figure 3A) and cell death (Figure 3B).

Results from our earlier studies indicate that the ratio of VDAC to HxK is a decisive factor of cell death.^18^ Much of the data underlying this conclusion came from the overexpression of one or other of the proteins. Here we studied the effect of reducing HxKII levels in the presence of endogenous VDAC levels. Loss of mitochondrial membrane potential in HeLa cells transfected with shHxKII (without heterologous expression of VDAC) were observed by using differential uptake of mitochondrial membrane potential-sensitive dye MitoTracker Red (MTR; Figure 3C). Most of the mitochondria in the HxKII-silenced cells show poor uptake of MTR, indicating loss of membrane potential as opposed to brightly stained mitochondria in the untransfected cells in the same field (Figure 3C) or cells transfected with control vector (Supplementary Figure S1C). Two VDAC inhibitors, DIDS and RuR, prevented the mitochondrial membrane potential loss induced by HxKII silencing (Figure 3C), thereby establishing that the effect of reducing HxKII levels on mitochondria was brought about through VDAC.

#### Endogenous HxKII level

**Figure 4:**
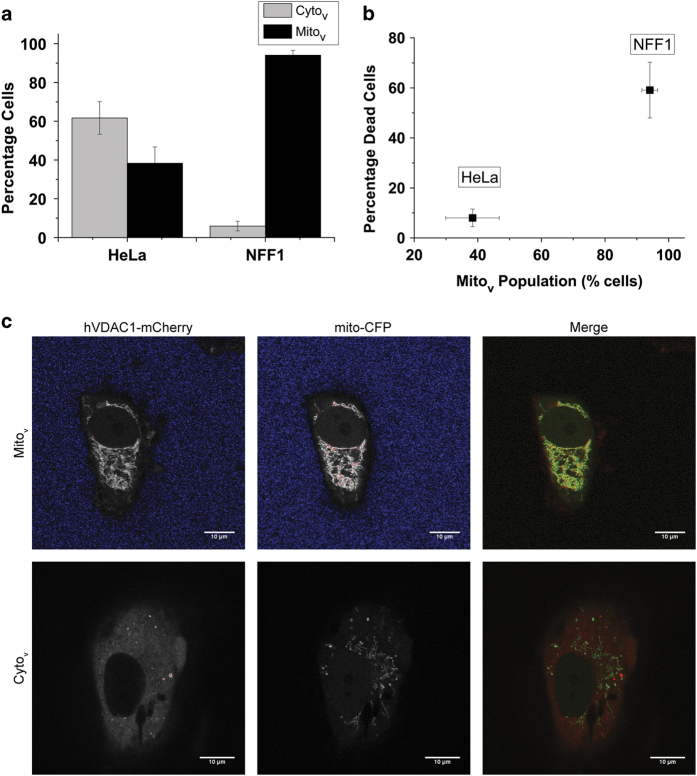
Endogenous levels of HxK influences VDAC localization and cell death. (**a**) Localization of hVDAC1-GFP 12 h after transfection into NFF1 or HeLa cells. (**b**) Percentage cell death as monitored by nuclear fragmentation in transfected NFF and HeLa cells with mitochondrially localized hVDAC1-GFP (Mito_v_). The analysis was carried out 12 h after transfection. Data represent mean and S.E of three experiments, with ≥100 cells scored in each experiment. (**c**) Representative images of NFF cells showing mitochondrial (upper panel) and cytosolic (lower panel) localization of hVDAC1-mCherry. Mitochondria are marked by mito-CFP.

Endogenous HxK levels vary across tissues and cell types. For example, elevated levels of HxKII have been reported as a hallmark of cancer cells.^10^ We used this fact to analyse the effect of endogenous HxK levels on VDAC localization and cell death. Western blottings reveal that the expression level of HxKII in primary neonatal foreskin fibroblast (NFF) cells is much lower than in the cervical cancer cell line HeLa (Supplementary Figures S2A and B). When transfected with hVDAC1-GFP, >90% of NFF cells fell in the Mito_v_ fraction as compared with only 40% in HeLa cells 12 h after transfection (Figures 4a and c). NFF cells showed >60% cell death as compared with ~10% in HeLa cells at this time point (Figure 4b). The large fraction of NFF cells undergoing cell death at 12 h precluded the possibility of extending the experiment to longer time points.

### Functional inhibition of HxKII

**Figure 5:**
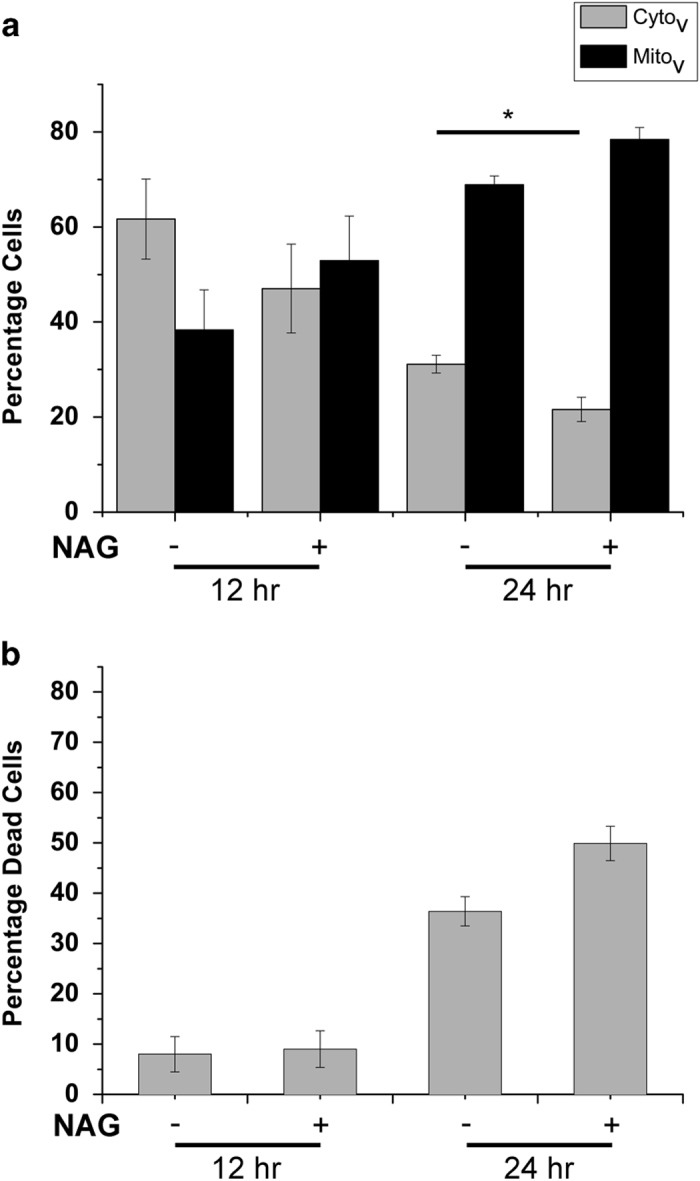
Effect of functional inhibition of HxK by NAG on localization of heterologously expressed hVDAC1-GFP and related cell death. HeLa cells transfected with hVDAC1-GFP were scored for (**a**) cytosolic or mitochondrial localization of the protein and (**b**) the percentage of cell death in the presence of absence of 1 mM NAG at 12 and 24 h after transfection. Data represent mean and S.E. of four experiments, with around 100 cells scored in each experiment. *P<0.01 based on Student’s unpaired *t*-test.

*N*-acetyl glucosamine (NAG), a glucose analogue, is a competitive inhibitor of HxK enzymatic activity. To test the hypothesis that functionally active HxK is required to modulate VDAC localization, hVDAC1-GFP-transfected HeLa cells were treated with 1 mM NAG. Twenty-four hours after transfection, the Cyto_v_ fraction showed a small but significant reduction in the NAG-treated group (Figure 5a). Cell death was up by a third compared with untreated cells at this time point (49% *versus* 36%, Figure 5b).

### Physical interaction of HXKII and VDAC

VDAC and HxK have been proposed to interact directly with each other.^2,10,32^ Specific regions or residues have been implicated in this interaction on the basis of mutations that perturb the extent of HxK modulation of VDAC function.^1,2^ The E72 residue in murine VDAC has been shown to be critical for interaction with HxK.^1,2^ We have mutated hVDAC1 at the equivalent position (hVDAC1-E73Q-GFP). In the case of HxKII, the N-terminal region (residues 1–15), which is required for its mitochondrial localization and interaction with VDAC,^10,31,33^ was deleted (Supplementary Figure S1C). These constructs were used in HeLa cells and their effect on VDAC localization and cell death was analysed.

#### Mutation in VDAC

**Figure 6:**
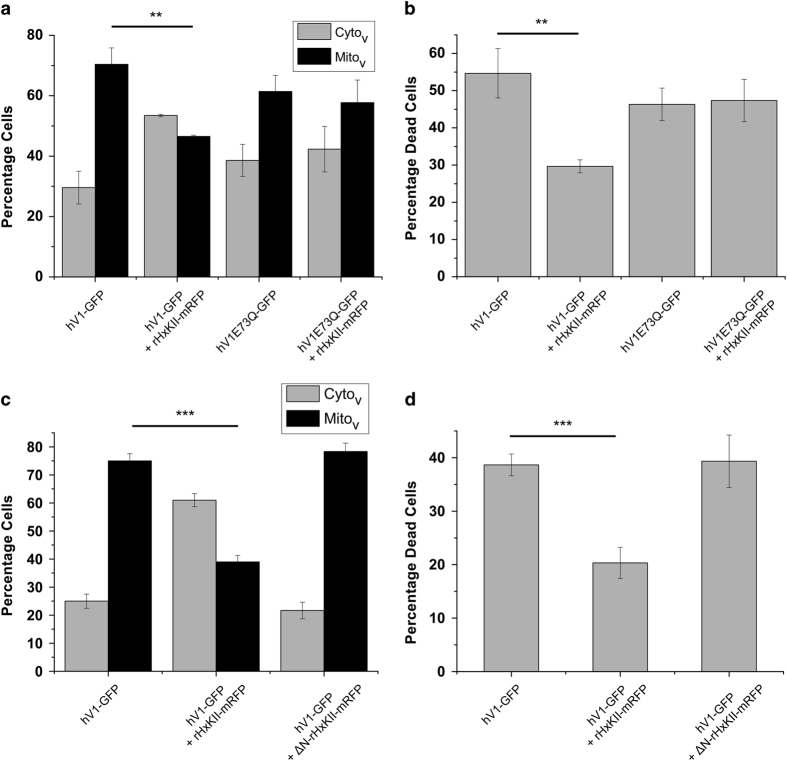
Effect of VDAC and HxKII mutations on HxKII-induced alterations in intracellular distribution of hVDAC1-GFP (hV1 GFP) and cell death in HeLa cells. (**a**) Distribution of hVDAC1-GFP in HeLa cells 20 h after transfection. The cells were cotransfected with rHxKII-mRFP and either wild-type hVDAC1-GFP or hVDAC1-E73Q-GFP. (**b**) Percentage cell death in the same experimental groups as in panel **a**. Data represent mean and S.E. of three independent experiments, with around 100 cells scored in each experiment. **P<0.005 based on Student’s unpaired *t*-test. (**c**) Distribution of hVDAC1-GFP in HeLa cells 20 h after transfection when coexpressed with wild-type (rHxKII-mRFP) or N-terminal deletion version of rHxKII (ΔN18-rHxKII-mRFP). (**d**) Percentage of cell death in the experimental groups from panel **c**. Data represent mean and S.E. of three experiments, with a minimum of 100 cells scored in each experiment. ***P=0.001 based on Student’s unpaired *t*-test.

When expressed in HeLa cells, localization of hVDAC1-E73Q-GFP and related cell death was comparable to that of hVDAC1-GFP. Forty-eight hours after transfection with hVDAC1-E73Q-GFP, 60% cells have Mito_v_ (Figure 6a) and 46% cells have fragmented nuclei (Figure 6b). However, unlike in the case of hVDAC1-GFP, localization of hVDAC1-E73Q-GFP and related cell death remained unaffected by coexpression of rHxKII-mRFP (Figures 6a and b).

#### HxKII N-terminal deletion

Overexpression of an N-terminal deleted form of HxKII (ΔN18-HxKII-mRFP) along with hVDAC1-GFP (Figures 6c and d) neither influenced mitochondrial localization of VDAC nor ameliorated the related cell death.

Thus mutations that have been reported to abrogate the HxK–VDAC interaction also abolish the ability of HxK to bias the cytoplasmic localization of VDAC and to attenuate death mediated by VDAC.

### Interaction of endogenous VDAC and HxK

VDAC is a mitochondrial protein that is encoded in the nuclear genome and, as such, is synthesized on cytoplasmic ribosomes and then translocates to mitochondria.

**Figure 7:**
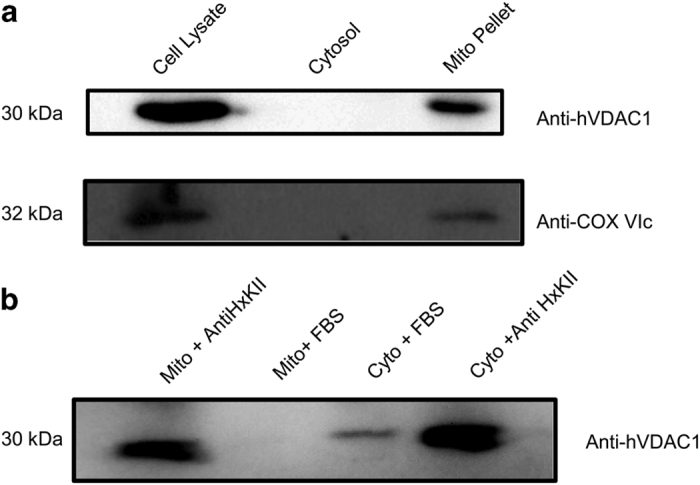
Interaction between endogenous VDAC and HxK (**a**) Western blotting analysis of different cellular fractions by using anti-hVDAC1 antibody. The samples are also probed with anti-COX VIc antibody as a marker for mitochondria to assess the purity of the fractions. (**b**) Western blotting analysis of samples immunoprecipitated with anti-HxKII antibody and probed with anti-VDAC antibody. Fetal bovine serum (FBS) is used as an antibody control. A band observed in cytosolic fraction with FBS indicates background, non-specific binding.

The observation that modulating endogenous HxKII affects localization of exogenous VDAC and cell death raises the possibility that the endogenous proteins may interact in the cytosol. To check this, cytosolic and mitochondrial fractions of HeLa cells were separated and subjected to pull down by an HxKII-specific antibody (Figure 7a) to look for interactions in either compartment.

Subcellular fractionation followed by immunoprecipitation of HxKII and complexes containing HxKII from a mitochondrial fraction resulted, unsurprisingly, in the pull down of VDAC. Surprisingly, a similar result was seen on immunoprecipitation of the cytosolic (100 000×*g* supernatant) fraction as well – indicating the presence of HxK-bound VDAC in the cytosol (Figure 7b).

## Discussion

VDAC, HxK and Bcl-2 family proteins have emerged as important regulators of cell death. VDAC has been shown to be a major player in the release of proteins normally resident in the mitochondrial intermembrane space to the cytosol, setting off an apoptotic cascade that results in cell death. This process can be attenuated by VDAC modulators, including antiapoptotic members of Bcl2-family proteins, HxK, DIDS and RuR.^2,21^ VDAC protein levels that deviate from the norm have been reported to perturb cellular homeostasis. For example, higher than normal expression of VDAC is observed in cancer^2,3^ and neurodegeneration^12^ while mitochondrial myopathy is associated with lower levels of expression.^34^ Our earlier study and those of others establishes that heterologous expression of VDAC results in cell death.^1,21^ A GFP-tagged version of rice VDAC (OsVDAC4-GFP) expressed in both mammalian (Jurkat) and plant (BY2) cells was found to be diffusely distributed through the cytoplasm of some cells while it localized to mitochondria in other cells. Clumping of mitochondria was observed in only those cells in which the protein was present in mitochondria. These cells exhibited symptoms of cell death, whereas cells where the protein was present exclusively in the cytoplasm appeared healthy.^18,21^ Further we have shown that, in addition to mitochondrial localization of VDAC, the ratio of VDAC to HxK expression also has a role in setting the level of cell death induced by VDAC overexpression.^18^ Here we have asked whether these two features – mitochondrial localization and cell death – are mechanistically linked.

We have focused on the consequences of HxK interacting with VDAC. We and others have previously shown that overexpression of HxK reduces the extent of cell death induced either by heterologous expression of VDAC or by stimuli such as staurosporin that utilize endogenous VDAC.^21^ We have tested the implied obverse – that reduction in the levels of HxKII alone would enhance cell death on VDAC expression. This was indeed the case when HxKII was silenced (Figure 3). Toxicity due to reduction in HxKII protein level is conceivably due to its homeostatic involvement in glycolysis. However, there are multiple isoforms of HxK, with HxKI also present in both cytosol and on the mitochondrion, suggesting redundancy. In addition, we should point out that knocking down HxKII affected localization of VDAC, with little or no effect on death.

An alternate way of looking at cells with differing amounts of HxK protein is to use cell lines with different levels of HxK expression. Cancer cells lines such as HeLa have high expression of HxK, while primary cultures express much lower levels of the protein. Experiments comparing HeLa and NFF cells leads to the same result, that is, the extent of VDAC toxicity is inversely correlated with HxKII levels (Figure 4). In every case, we also observed that the extent of VDAC toxicity is correlated with the extent of its localization to mitochondria.

Sun *et al.*^31^ have previously shown that the full extent of the antiapoptotic activity of HxK requires both its N-terminus and its glucose-phosphorylating enzymatic activity. The NAG experiment effectively reproduces the effect of loss of enzymatic activity. At the concentrations used, treatment with NAG has a small but significant effect on the distribution of heterologously overexpressed VDAC as well as cell death (Figure 5). Most tellingly, an N-terminal deletion of HxKII^33^ and mutations in VDAC that abrogated the VDAC–HxKII interaction rendered both mitochondrial localization of VDAC and its toxicity independent of the level of HxKII expression (Figure 6). This indicates that not only attenuation of cell death but also VDAC retention in the cytosol requires physical interaction with HxKII.

**Figure 8:**
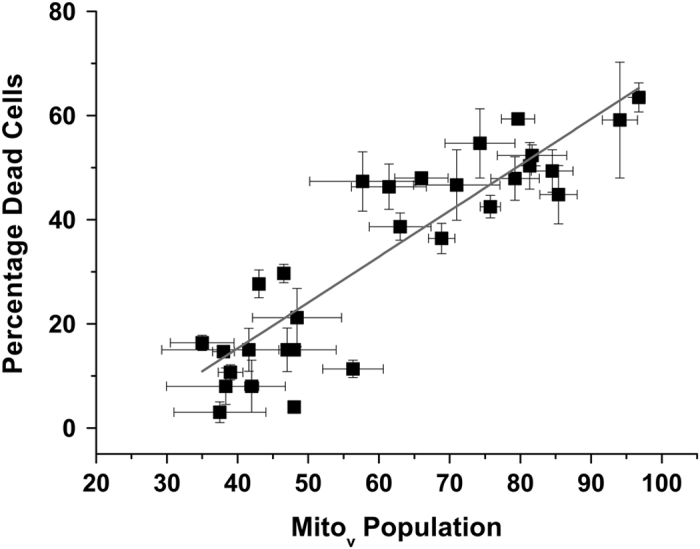
Correlation between mitochondrial localization of heterologously expressed VDAC and cell death. The best-fit line has an *R* value of 0.81.

VDAC is a mitochondrial protein that is encoded in the nuclear genome and as such is synthesized on cytoplasmic ribosomes and then translocated to mitochondria. All previous reports of the modulation of VDAC-induced cell death by cytoplasmic proteins have utilized heterologously expressed VDAC protein. Heterologous expression of VDAC results in a bolus of protein being synthesized on cytoplasmic ribosomes. Our results show that heterologously expressed VDAC remains in the cytosol for some time before being incorporated into the mitochondrion.^21^ Additionally, our current data emphasize a strong correlation between VDAC incorporation into mitochondria on the one hand and cell death on the other (Figure 1). We have shown that two antiapoptotic proteins, Bcl2 and HxK, significantly extend the cytosolic residence time of heterologously expressed VDAC protein (Figure 2). The good correlation between changes in distribution pattern of VDAC protein (cytosol *versus* mitochondria) and cell death in the presence of physiological partners such as Bcl2 and HxK suggests retention of VDAC in the cytosol as a novel mechanism for modulating cell death (Figure 8).

The structure of cytosolic VDAC is open to conjecture and it is conceivable that it may differ from the *β* barrel seen in the crystal and NMR structures and presumably present in membranes. If that were so, the accessibility of residues such as E72 to reagents as large as HxK may not be limiting in the cytosol. Indeed our localization studies suggest strongly that interaction with E73 of hVDAC1 is critical to its cytosolic retention by HxKII (Figure 6a). Once on the mitochondrion, however, additional interacting sites may come into play.

VDAC is often used as a marker for mitochondria on western blottings as nearly all of the endogenous protein is mitochondrially localized. It may thus be presumed that no endogenous VDAC is present in the cytosol, thereby limiting this mechanism of modulation of cell death to artefactual overexpression of heterologous protein. We also found that no VDAC protein could be detected on the western blotting of ~50 *μ*l of the cytosolic fraction (Figure 7a). However, all our results lead to the piquant suggestion that there may be a small but significant pool of endogenous VDAC in the cytosol possibly in a complex that includes HxK. Indeed, in normal HeLa cells, on using an anti-HxK antibody for immunoprecipitation from a volume of 2 ml of cytosol and then probing a western blotting with anti-VDAC antibody, we did detect VDAC protein (Figure 7b). The pull down clearly concentrated cytosolic VDAC to detectable levels. More importantly, it revealed that cytosolic VDAC is associated with HxK in a stable complex under endogenous conditions. It is not implausible that delivery of freshly synthesized VDAC to the mitochondrion may be regulated in a manner that involves HxK.

It may be noted that fine tuning of the levels of proteins on the plasma membrane can have consequences ranging from the time course of action potentials to cell survival and death. For instance, the tumour-suppressor KCNRG appears to function by retaining potassium channel Kv1 proteins in the endoplasmic reticulum.^35^ Thus posttranslational regulation of the density of specific proteins in their target membranes appears to be critical.

The results from this study indicate that some of the dramatic effects of HxK in attenuating cell death induced by heterologously expressed VDAC arise from retention of the channel protein in the cytosol. HxK and Bcl2 thus appear to exert antiapoptotic effects in at least two different ways – by retaining the protein in the cytosol and by specific interactions on the mitochondrion. Perturbation of the interactions that lead to cytosolic retention could thus modulate the propensity of a cell to undergo apoptosis in response to external stimuli and hence sensitivity to drugs that induce cell death.

## Materials and methods

### Cell lines, transfection and imaging

Primary NFF cells and HeLa cell line were gifts from Professor MM Panicker and Professor Apurva Sarin (NCBS, Bangalore, India), respectively. Both cell lines were grown in a humidified atmosphere of 95% air and 5% CO_2_ in DMEM with 2 mM L-glutamine supplemented with 10% FBS, together with a mixture of 1000 U/ml penicillin and 1 mg/ml streptomycin. For transient transfections, HeLa cells were grown to 50% confluency in 35 mm coverslip dishes and transfected using Effectene (Qiagen, Hilden, Germany) according to the manufacturer’s protocol. For each transfection, 100 ng of plasmid DNA was used. Imaging and cell counting were carried out at specified time points.

Transient transfection of NFF cells was carried out by electroporation. The cells were grown in culture flasks with 25 cm^2^ surface area to 80% confluency and then harvested using trypsin treatment. Cells were kept at 4 °C for 10 min in serum-free DMEM with appropriate DNA (1 *μ*g) and then electroporated with a single square wave pulse of 250 V for 20 ms using Bio-Rad micropulser II (Bio-Rad, Hercules, CA, USA). Cells were kept on ice for 10 min after electroporation and then suspended in DMEM with 10% FBS.

Laser scanning confocal microscopy (Olympus FV1000, Olympus Corporation, Shinjuku, Tokyo, Japan) was used for subsequent imaging. Lasers and filters used are described for each set of experiments in the respective Results section.

### Plasmids and site-directed mutagenesis

Human VDAC1 (hVDAC1) cDNA was a kind gift from Professor Mike Forte (Oregon Health and Science University, Portland, OR, USA). The hVDAC1 insert was subcloned into the NheI and XhoI restriction sites of plasmid pEGFP-N1 to generate a green fluorescent protein-tagged version, hVDAC1-GFP. The red fluorescent protein mCherry gene was amplified from mCherry-pAN583 plasmid (a kind gift from Professor Andreas Nebenfuhr, University of Tennessee, Knoxville, TN, USA) using the forward primer 5′- CGG**GGTACC**CCGGATGGTGAGCAAGGG-3′ and the reverse primer 5′- GCTCTAGAGCTTA**AGATCT**GTACAGCTCGTCCATGCCGCCG-3′. The amplicon was digested with XbaI and KpnI and swapped in for GFP in hVDAC1-GFP digested with the same enzymes. Red fluorescent protein-tagged constructs of Rat HxKII (rHxKII-mRFP) and human Bcl2 (Bcl2-mRFP) were gifts from Professor Apurva Sarin (NCBS, Bangalore, India). Silencing constructs for HxKII along with their control plasmids were purchased from Origene, Rockville, MD, USA.

Site-directed mutagenesis in hVDAC1 and rHxKII was carried out by a PCR-based method. hVDAC1 (E73Q) was constructed by using the forward primer 5′- CGGCCTGACGTTTACA**CAG**AAATGGAATAC-3′ and the reverse primer 5′- CGGTATTCCATTT**CTG**TGTAAACGTCAGG-3.' ΔN18-rHxKII was constructed by a two-step site-directed mutation in rHxKII gene. First, two methionine residues, M1 and M4, were replaced by Leucine residues to remove the first two start codons. The primers used were forward primer 5′- CGAGCTCAAGCTTCGAATTC**CTG**ATCGTTTCGCAT**CTG**ATCGCCTGC-3′ and reverse primer 5′- GAATAAGCAGGCGATCA**GAT**GCGAAACGATCA**GGAA**TTCGAAGCTTG-3′. Further, a start codon was added at position 18 of the earlier construct by converting valine to methionine with the forward primer 5′- CGGAGCTCAACCAAAACCAA**ATG**CAGAAGGTTGACC-3′ and the reverse primer 5′- GAAATTGGTCAACCTTCTG**C**ATTTGGTTTTGGTTGAGC-3′ to get 18 M rHxKII, which is called ΔN18-rHxKII.

### Analysis and quantification of intracellular distribution of hVDAC1

To determine the intracellular localization of VDAC, cells were cotransfected with hVDAC1-GFP/hVDAC1-mCherry and plasmid with mitochondria-targeted cyan fluorescent protein (mito-CFP; a kind gift from Dr V Sriram, formerly in NCBS, Bangalore, India). Doubly transfected cells were observed and imaged at different time points after transfection by using appropriate wavelengths (For GFP, *λ*_ex_ 488 nm and *λ*_em_ 510–540 nm; for CFP, *λ*_ex_ 405 nm and *λ*_em_ 465–485 nm; for mCherry, *λ*_ex_ 543 and *λ*_em_ 600–680 nm). Uniform distribution of GFP/mCherry fluorescence was taken as diagnostic of cytosolic localization, whereas punctate patterns overlapping with mito-CFP indicated a mitochondrial localization. In the case of cells transfected with rHxKII-mRFP or Bcl-2-mRFP, red fluorescence was observed and imaged with *λ*_ex_ 543 nm and *λ*_em_ 570–650 nm.

### Quantification of cell death

Cell death was quantified as described in Godbole *et al.*^21^ using nuclear fragmentation as the indicator. Cells were stained with Hoechst-33342 (1 *μ*M) for 15 min and washed with 1× PBS. The number of cells with fragmented nuclei was counted using UV filter settings (*λ*_ex_ 350 nm and *λ*_em_ 465–485).

### Inhibition of HxK function

To inhibit HxK function, HeLa cells were treated with 1 mM NAG for 1 h before transfection with VDACs. Cells were then maintained in 1 mM NAG for the duration of the experiment. Transfected cells were imaged 12 and 24 h after transfection and quantitated.

### Silencing of HxKII expression

For silencing the HxKII expression using RNAi constructs, HeLa cells were grown to 60–70% confluency and then transfected with silencing constructs or scrambled plasmid along with hVDAC1-mCherry. Silencing of HxKII in HeLa cells was confirmed by western blotting with anti-HxKII antibody (1 : 1000). Transfected cells were quantified and imaged 20 h after transfection for localization of hVDAC1-mCherry.

In cells with reduced expression of HxKII, the effect of VDAC inhibition was studied by treating the cells with DIDS or RuR. HeLa cells were grown to 60–70% confluency and then treated with fresh DMEM or with DMEM containing DIDS (50 *μ*M)/RuR (1 *μ*M) for 1 h. The control or DIDS/RuR-treated cells were then transfected with HxKII-silencing construct (mentioned above) or with control plasmid. Cells were further grown in the control media or media containing DIDS/RuR. Twenty-four hours after transfection, cells were treated with 1 *μ*M of MTR in 1× PBS for 20 min at RT. Cells were washed with 1× PBS and then imaged by using appropriate wavelengths (For GFP, *λ*_ex_ 488 nm and *λ*_em_ 510–540 nm; and for MTR, *λ*_ex_ 543 and *λ*_em_ 600–680 nm).

### Gel electrophoresis and western blotting analysis

Polyacrylamide gel electrophoresis (SDS-PAGE) was performed according to Laemmli.^36^ For VDAC and HxKII proteins, we have used 12% and 8% SDS-PAGE, respectively. Gels were electroblotted on to PVDF membrane (Pall Life Sciences, Port Washington, NY, USA) and immunostained with monoclonal anti-VDAC1, polyclonal anti-HxKII and monoclonal anti-*β*-tubulin followed by HRP-conjugated secondary anti-mouse IgG for anti-VDAC and anti-*β*-tubulin and anti-rabbit IgG for anti-HxKII.

### Mitochondria isolation and immunoprecipitation

Pure mitochondria and cytosol were isolated from HeLa cells by using a protocol modified from Arnoult *et al.*^37^ Briefly, cells were grown to 80% confluency, harvested and collected by centrifugation at 1200×*g* for 8 min at 4 °C and washed twice with 1× PBS at the same speed. Cells were incubated for 20 min in MB buffer (210 mM mannitol, 10 mM HEPES pH-7.5, 0.1 mM EGTA and 1× protease inhibitor cocktail). After incubation, cells were homogenized with a Dounce homogenizer (30 strokes). Homogenized samples were centrifuged at 500×*g* for 8 min at 4 °C to pellet cell debris. The supernatant S1 was re-centrifuged at 2000×*g* for 15 min at 4 °C to pellet nuclei and unbroken cells. The supernatant S2 was then centrifuged at 15 000×*g* for 30 min at 4 °C to pellet mitochondria, and the supernatant S3 was stored. The mitochondrial pellet was resuspended in MB buffer and washed twice with MB buffer. The final mitochondrial pellet was suspended in IP buffer (50 mM Tris pH-7.5, 150 mM NaCl, 0.1 mM EDTA and 1× protease inhibitor cocktail). Supernatant S3 was centrifuged twice at 120 000×*g* for 30 min at 4 °C, and the final supernatant collected as a pure cytosolic fraction. Total protein was quantified using the BCA method (Bangalore Genei India, Bangalore, India).

Immunoprecipitations were carried out using protein A/G-coated agarose beads (Santa Cruz, Santa Cruz, CA, USA) following the manufacturer’s instructions. In all, 20 *μ*l of protein A/G agarose beads was blocked using 1 ml of 1% BSA in 1× PBS for 1 h prior to immunoprecipitation. Blocked beads were washed thrice with 1× PBS at 5000×*g* for 5 min each. The resulting bead pellets was resuspended with either FBS or primary anti-HxKII antibody and incubated overnight at 4 °C on a rocker. The next day, the beads–antibody complex was pelleted at 5000×*g* for 5 min. Pellets were washed three times with 1× PBS at the same speed and time. Mitochondrial and cytosolic fractions were separately incubated with the beads–antibody complex for 2 h at 4 °C. Immunoprecipitated products were pelleted at 1000×*g* for 5 min at 4 °C. Pellets were washed with 1× PBS seven times at the same speed and time. After the final wash, pellets were resuspended in 40 *μ*l of SDS loading buffer. Samples were heated at 60 °C for 30 min and then loaded in 8% SDS gel for western blotting.

## Abbreviations

VDAC: voltage-dependent anion channel
HxK: hexokinase
RuR: Ruthenium Red
DIDS: 4,4′-diisothiocyanostilbene-2,2′-disulfonic acid
MTR: MitoTracker Red.
